# Bactericidal Activity of the Human Skin Fatty Acid *cis*-6-Hexadecanoic Acid on Staphylococcus aureus

**DOI:** 10.1128/AAC.01043-13

**Published:** 2014-07

**Authors:** Michaël L. Cartron, Simon R. England, Alina Iulia Chiriac, Michaele Josten, Robert Turner, Yvonne Rauter, Alexander Hurd, Hans-Georg Sahl, Simon Jones, Simon J. Foster

**Affiliations:** a The Krebs Institute, Department of Molecular Biology and Biotechnology, University of Sheffield, Western Bank, Sheffield, United Kingdom; b Department of Chemistry, University of Sheffield, Brook Hill, Sheffield, United Kingdom; c Institute of Microbiology, Immunology and Parasitology, Medical Faculty, University of Bonn, Bonn, Germany

## Abstract

Human skin fatty acids are a potent aspect of our innate defenses, giving surface protection against potentially invasive organisms. They provide an important parameter in determining the ecology of the skin microflora, and alterations can lead to increased colonization by pathogens such as Staphylococcus aureus. Harnessing skin fatty acids may also give a new avenue of exploration in the generation of control measures against drug-resistant organisms. Despite their importance, the mechanism(s) whereby skin fatty acids kill bacteria has remained largely elusive. Here, we describe an analysis of the bactericidal effects of the major human skin fatty acid *cis*-6-hexadecenoic acid (C6H) on the human commensal and pathogen S. aureus. Several C6H concentration-dependent mechanisms were found. At high concentrations, C6H swiftly kills cells associated with a general loss of membrane integrity. However, C6H still kills at lower concentrations, acting through disruption of the proton motive force, an increase in membrane fluidity, and its effects on electron transfer. The design of analogues with altered bactericidal effects has begun to determine the structural constraints on activity and paves the way for the rational design of new antistaphylococcal agents.

## INTRODUCTION

The human skin provides important protection against microbial infection. The epidermis provides a physical barrier via an external cross-linked keratin layer upon a scaffold of keratinocytes. Most importantly, the skin also has a broad range of innate immunity components, including antimicrobial peptides and fatty acids (1, 2, 3). Despite these properties, the skin is host to a large and complex microflora of >1,000 species (4). Many of these microorganisms are harmless commensals, but there are also opportunist pathogens. Thus, how the skin is able to defend against potentially dangerous organisms is of great interest, as harnessing our innate defenses may lead to novel control regimens.

Staphylococcus aureus is an extremely versatile bacterium, whose primary niche is as a commensal in the human nose. Despite being carried harmlessly by 30% of the human population, this organism is an opportunist pathogen causing many infections and deaths worldwide (5). The problem is exacerbated by the alarming spread of antibiotic resistance; in particular, methicillin-resistant S. aureus (MRSA) is prevalent in hospitals and is beginning to spread in the wider community. The ability of S. aureus to survive in the nares and on the skin is an important facet of its capacity to spread from host to host. In particular, skin fatty acids in sebum have been found to be potent staphylocidal agents (3, 6, 7). Fatty acids are an important facet of our innate defenses, and in fact, a Toll-like receptor-mediated pathway in mice leads to increased fatty acid production and protection against S. aureus skin infections (8). The most important antistaphylococcal human skin fatty acid is *cis*-6-hexadecanoic acid (C6H) (3, 7). Patients with atopic dermatitis show reduced C6H levels and increased colonization with S. aureus, and in these patients topical treatment with C6H results in a decrease in S. aureus levels (7). We have also shown that purified C6H treats both cutaneous and systemic models of S. aureus disease (9). Fatty acids also kill S. aureus within abscesses (10). As well as being bactericidal, human sebum and C6H at sublethal concentrations inhibit the production of virulence determinants and the induction of antibiotic resistance by S. aureus and other important pathogens (9). Thus, fatty acids can debilitate potentially harmful bacteria at several levels.

In response to such a potent molecule, S. aureus possesses a number of resistance mechanisms, which allow it to withstand skin fatty acids (9, 11). We have found that the major S. aureus surface protein, IsdA, is produced in response to the lack of available iron associated with the human host and is required for nasal colonization (9). IsdA contributes to skin fatty acid resistance by rendering the cells more hydrophilic via its C-terminal domain. It is also this domain that is required for survival of S. aureus on human skin. Thus, the interaction between S. aureus and human skin fatty acids is a crucial factor in its ability to colonize a host. Despite the importance of this ability, the bactericidal mechanism of action of skin fatty acids on S. aureus is still unknown. The surfactant nature of these compounds likely results in membrane perturbation. Fatty acids also inhibit many central metabolic processes, but this may occur indirectly via uncoupling of ATP synthesis (12, 13). In addition, the accumulation and incorporation of linoleic acid (12, 14) may result in toxic lipid hydroperoxides (15, 16). In this study, we aimed to elucidate the mode of action of the major staphylocidal skin fatty acid, C6H, on S. aureus. This revealed multiple mechanisms of killing via the ability of C6H to disrupt essential membrane functions.

## MATERIALS AND METHODS

### Bacterial strains and chemicals.

S. aureus strain SH1000 was used in all assays except as otherwise stated. Inverted vesicles and membranes were prepared from *Micrococcus flavus* and Escherichia coli K-12. All chemicals were purchased from Sigma-Aldrich except as otherwise stated. Radiolabeled [^14^C]UDP *N*-acetylglucosamine (UDP-GlcNAc) was from Hartmann Analytic (Germany) and undecaprenyl phosphate (C55-P) was from Larodan Fine Chemicals (Sweden). Bacteria were routinely grown in brain heart infusion (BHI) medium aerobically; for anaerobic growth, cells were grown in airtight anaerobic jars.

### C6H killing assays.

Bacteria were grown to an optical density at 600 nm (OD_600_) of ∼0.6 in iron-limited tryptic soy broth (TSB) or chemically defined medium (CDM) (17). Cells were harvested by centrifugation at 3,000 × *g* (at 4°C for 5 min) and washed twice in sterile distilled water (dH_2_O) by centrifugation and resuspension. Cell suspensions (∼2 × 10^8^ CFU/ml in appropriate buffers) were incubated at 37°C with and without C6H (and other chemicals). Except when stated otherwise, all experiments were done in 20 mM morpholineethanesulfonic acid (MES) (pH 5.5) with 3 μg/ml C6H. Cell viability was determined by plating on TSB agar (9).

### MICs.

MICs were determined as previously described (9).

### Assessment of membrane integrity.

Membrane integrity was assayed by determination of the permeability of the cells to propidium iodide (PI). Nisin served as a positive control for membrane disruption. Bacteria were prepared as for the C6H killing assay (in 20 mM MES) and PI was added to the cell suspension to a final concentration of 13 μM. Fluorescence of the mixture was followed with excitation at 535 nm and emission at 617 nm (18). After a 1-min equilibration time, C6H was added to the assay (at 3 or 5 μg/ml).

### Effect of C6H on lipid II polymerization.

Lipid II was purified as described by Schneider et al. (19). The enzymatic activity of S. aureus penicillin-binding protein 2 (PBP2) was determined by incubating 2.5 nmol lipid II in 100 mM MES, 10 mM MgCl_2_ (pH 5.5), and 0, 2, 4, 20, and 40 nM C6H in a total volume of 50 μl. The reaction was initiated by the addition of 7.5 μg PBP2-His_6_ and incubated for 1.5 h at 30°C. Residual lipid II was extracted from the reaction mixtures with *n*-butanol-pyridine acetate (pH 4.2) (1:1, vol/vol), and analyzed by thin-layer chromatography (TLC) (silica plates [60F254]; Merck) using chloroform-methanol-water-ammonia (88:48:10:1, vol/vol) as the solvent (20). Spots were visualized by phorbol myristate acetate (PMA) staining reagent (phosphomolybdic acid 2.5% [vol/vol], ceric-sulfate 1% [wt/vol], and sulfuric acid 6% [vol/vol]). After the TLC plate was dried, spots were developed by heating at 150°C (21).

### Inhibition of *in vitro* lipid II synthesis.

Inhibition of lipid II synthesis was performed *in vitro* using membrane preparations of Micrococcus luteus DSM 1790 as described by Schneider et al. (19) with the addition of radiolabeled [^14^C]UDP-GlcNAc. Membranes were isolated from lysozyme-treated cells by centrifugation (40,000 × *g* for 60 min at 4°C), washed twice in 50 mM Tris-HCl and 10 mM MgCl_2_ (pH 7.5), and stored under liquid nitrogen until use. Reaction mixtures were carried out in a final volume of 75 μl and contained 400 μg of membrane protein, 5 nmol undecaprenyl phosphate (C55-P), 50 nmol UDP-*N*-acetylmuramyl pentapeptide (UDP-MurNAc-PP), 50 nmol [^14^C]UDP-GlcNAc in 60 mM Tris-HCl (pH 8), 5 mM MgCl_2_, and 0.5% (wt/vol) Triton X-100. UDP-MurNAc-PP was purified as described previously (22). C6H was added to the reaction mixture in molar ratios of 2:1 (referring to the total amount of C55-P [5 nmol]). After 1 h at 30°C, the lipids were extracted with 1 volume of *n*-butanol-6 M pyridine-acetate (2:1, vol/vol) (pH 4.2). The reaction products were separated by TLC (silica plates [60F254]; Merck) using chloroform-methanol-water-ammonia (88:48:10:1) as the solvent (20). Radiolabeled spots were visualized using a biomolecular imager for radioisotope detection (Storm 820 PhosphorImager; Amersham Biosciences) and the image was analyzed with ImageQuant TL v 2005 (Nonlinear Dynamics, Ltd.) software.

### Determination of membrane potential using tetraphenylphosphonium ion (TPP^+^).

To 5 ml of cell suspension (prepared as described above) at 37°C, 5 μl of [^3^H]tetraphenylphosphonium ([^3^H]TPP) (final concentration, 1μCi/ml [0.74 to 1.48 TBq/mmol]) was added. One-hundred-microliter samples were removed, washed with phosphate-buffered saline (PBS) by filtration, and used to calculate total emission. Where appropriate, valinomycin and/or C6H was added (20 and 3 μg/ml final concentrations, respectively), and samples were prepared as described above. Calculation of the membrane potential (ΔΨ) was performed as previously described (23).

### Assessment of intracellular pH.

The experiments and controls were performed as previously described by Breeuwer et al. (24) using cells prepared as described above.

### ATP assays.

Cell suspensions were prepared as described above. Samples (200 μl) were taken and 800 μl dimethyl sulfoxide (DMSO) was added. Cells were recovered by centrifugation (10,000 × *g* for 5 min at room temperature [RT]), and the supernatant was removed for analysis of extracellular ATP. ATP levels were measured using a bioluminescence assay (Sigma).

### Respiration.

Cells grown as above for the killing assays were resuspended to an OD_600_ of ∼1.0 in iron-limited TSB and transferred to a Clark-type oxygen electrode chamber, and oxygen consumption was recorded.

### Membrane fluidity.

Cell membrane fluidity was determined by measuring fluorescence polarization with 1,6-diphenyl-1,3,5-hexatriene (DPH) (Sigma), as described by Bayer et al. (25). A 1-ml S. aureus cell suspension was prepared as described above (OD_600_ of ∼1.0) and DPH (2 μM final concentration) was added. Fluorescence polarization was measured immediately after addition of inhibitor to the labeled sample (1 ml) using an Edinburgh Instruments 199 spectrometer. The excitation and emission wavelengths were 360 and 426 nm, respectively. The degree of fluorescence polarization, or the polarization index, was calculated with the formula [*I_V_* − *I_H_* (*I*_HV_/*I*_HH_)]/[*I_V_* + *I_H_* (*I*_HV_/*I*_HH_)], where *I* is the corrected fluorescence intensity and the subscripts *V* and *H* indicate the values obtained with vertical or horizontal orientation of the analyzer, respectively. The lower the polarization index value, the more fluid the membrane (25, 26, 27).

### Iodonitrotetrazolium chloride reduction assay.

Reduction of the iodonitrotetrazolium chloride (INT) by the components of the electron transport chain (ETC) was performed as described by Smith and McFeters (28) in a 250-μl volume reaction.

### Preparation of inverted vesicles.

Inverted vesicles were prepared using a method adapted from Burstein et al. (29). Cells from a 1-liter TSB post-exponential-phase culture (OD_600_, 3 to 4) were harvested by centrifugation (3,000 × *g* for 10 min at 4°C) and washed with 100 ml buffer containing 50 mM Tris-HCl (pH 8.0), 2 mM MgCl_2_, 0.5 mM dithiothreitol, and 0.5 mM EDTA. The pellet was resuspended with 5 ml of the same buffer, the pH was adjusted to 8.0, and 10 μg/ml DNase and 10 μg/ml RNase were added. The cells were disrupted by mechanical lysis using a microorganism lysing kit (0.1-mm glass beads in 2-ml standard tubes) in a Precellys 24 homogenizer. The crude homogenate was centrifuged at 30,000 × *g* (for 20 min at 4°C). The resulting supernatant was centrifuged at 175,000 × *g* (for 120 min at 4°C) and the pellet containing the vesicles was finally resuspended in 2 ml of the above buffer supplemented with 10% (vol/vol) glycerol and stored in liquid nitrogen.

### Liposomes.

Liposomes were prepared and quantified according to Bonelli et al. (30). We dried 2 μmol 1,2-dioleoyl-*sn*-glycero-3-phosphocholine (DOPC) (Avanti Polar Lipids) in a glass tube and then added 300 μl of a solution of 50-mM carboxyfluorescein and 50 mM NaCl or KCl to the dried DOPC. The glass tube was vortexed thoroughly, freeze-thawed 10 times, and then extruded through a 400-nm pore membrane. Liposomes were loaded on a Sephadex G50 column preequilibrated with the appropriate buffer and harvested by centrifugation (13,000 × *g* for 1 min at RT).

### Production of C6H analogues.

The synthesis of C6H analogues and Bodipy dyes were carried out by sequential alkylation of alkynyl anions with appropriate alkyl and tetrahydropyranyl alkyl bromides, followed by dichromate oxidation to the carboxylic acid. Comprehensive details will be reported in due course. A method describing a similar strategy to produce related compounds has recently been described in the literature (31).

### Transmission electron microscopy.

Samples were prepared and analyzed as previously described (32).

### Fluorescence microscopy.

We added 17 μM Bodipy 1 (in DMSO) and 10 μl of a 1-μg/ml solution of Bodipy-labeled vancomycin (vancomycin, Bodipy FL conjugate; Invitrogen) to 1 ml of S. aureus (grown as above) and incubated the mixture for 5 min at 37°C on a rotary wheel (20 rpm). The cells were harvested by centrifugation (10,000 × *g* for 1 min at 4°C), washed 3 times by resuspension in dH_2_O, fixed, mounted, and examined by deconvolution microscopy as previously described (33).

### Ion level determination.

Cells were grown and treated as for the killing assays described earlier. At each time point, required cells were harvested by centrifugation (10,000 × *g* for 1 min at 4°C) and ion levels within the supernatant were monitored via the ion chromatography service (Kroto Centre, University of Sheffield).

### Statistics.

Student's *t* test was used where appropriate.

## RESULTS

### Characterization of the bactericidal effects of C6H.

**FIG 1:**
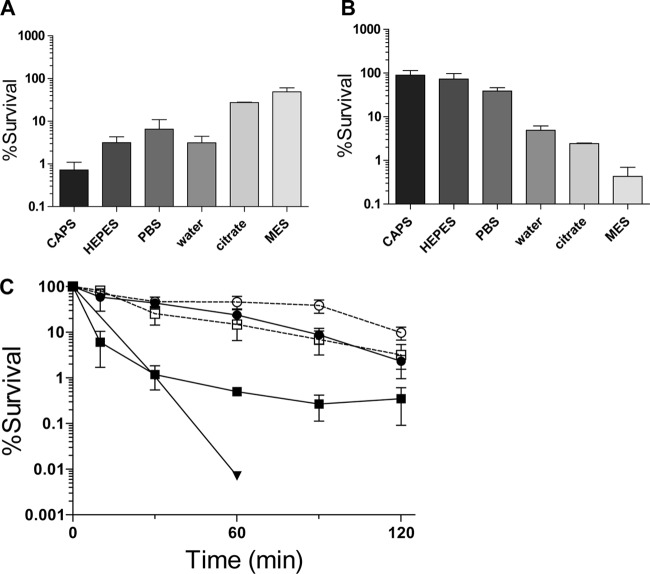
Characterization of the bactericidal effect of C6H. Effect of external pH on bactericidal activity. S. aureus cells were incubated (for 2 h at 37°C) in various buffers (20 mM *N*-cyclohexyl-3-aminopropanesulfonic acid [CAPS; pH 10], 25 mM HEPES [pH 7.4], 20 mM PBS [pH 7.4], 20 mM sodium citrate buffer [pH 5.5], and 20 mM MES [pH 5.5]). (A) Percentage of surviving cells with no C6H compared to the percentage of cells at 0 min. (B) Percent survival of the cells after incubation with 5 μg/ml C6H compared to percent survival of the cells with no C6H at the same time point. (C) Survival kinetics and the effect of KCl on C6H activity. The effect of 3 and 5 μg/ml C6H (● and ■, respectively) with (open symbols) or without (filled symbols) 50 mM KCl (▼, 10 μg/ml C6H). For all data points, the percent survival was compared to that of the wild type at the same time.

Previous work has demonstrated that C6H kills S. aureus (7, 9). It is well established that the pH of the skin is slightly acidic (34) and so the effect of pH on C6H sensitivity was tested (Fig. 1A). S. aureus shows greatest survival at pH 5.5 (∼skin pH) without C6H, losing only 50% viability after 2 h. Paradoxically, 5 μg/ml C6H has its greatest activity at pH 5.5, with only 0.5% survival after a 2-h treatment in MES buffer (Fig. 1B). There is also a clear C6H dose dependence in S. aureus survival at pH 5.5 (Fig. 1C). At 10 μg/ml, C6H viability is quickly lost (>99% death after 30 min). At 3 and 5 μg/ml C6H, 60 and 99% of cells were nonviable after 30 min, respectively.

**FIG 2:**
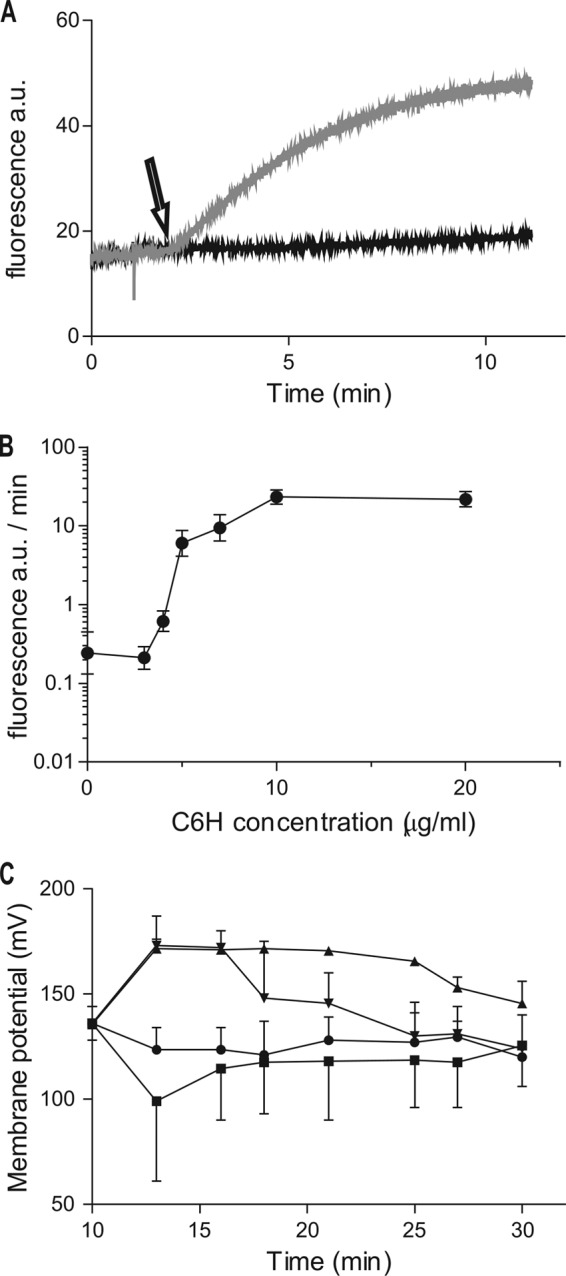
Effect of C6H on membrane integrity (A and B) and potential (C). (A) Cells were incubated with PI for 1 min and 20 mM MES before addition (black arrow) of 3 μg/ml C6H (black curve) and 5 μg/ml C6H (gray curve). (B) Effect of C6H on the rate of PI fluorescence increase. (C) Membrane potential was measured for 10 min before (at 0 min) the addition of 3 μg/ml C6H (■), 20 μg/ml valinomycin (▲), 3 μg/ml C6H, or 20 μg/ml valinomycin (▼) and after no addition (●).

In order to begin to determine the molecular mechanism of C6H bactericidal activity, propidium iodide (PI) was used. PI fluorescence greatly increases upon binding to nucleic acids and so it can be used as a marker for membrane integrity when added to the extracellular milieu. At pH 5.5, only concentrations of C6H above 3 μg/ml led to a rapid loss of membrane integrity, as shown by an increase in fluorescence (Fig. 2A and B). However, at 3 μg/ml C6H the cells still died. This suggests that death can occur independently of drastic membrane permeabilization.

### Inhibition of C6H activity by salts.

Potassium ions have previously been shown to affect fatty acid activity on mitochondria (35). Addition of 50 mM KCl to 50 mM MES (pH 5.5) gave a significant reduction in the rate of killing by 5 μg/ml C6H (*P* = 0.03 at 60 min) but to a lesser extent by 3 μg/ml C6H (*P* = 0.19 at 60 min) (Fig. 1C). Addition of NaCl or CaCl_2_ had a similar inhibitory effect (data not shown). Salt inhibition of C6H activity further suggests more than one mode of killing, disruption of membrane integrity and an uncharacterized mechanism at lower concentrations (≤3 μg/ml). The binding of PI to nucleic acid was inhibited in the presence of salt, hence preventing the fluorescence assay.

### Does C6H affect peptidoglycan synthesis?

Previously, the lantibiotic nisin has been shown at low concentrations to associate with lipid II (a precursor in the peptidoglycan biosynthesis pathway), which leads to simultaneous inhibition of cell wall biosynthesis and formation of defined pores in the cell membrane. At high concentrations it has several other effects, such as induction of autolysis and destabilization of the lipid bilayer (36). Initially, using M. luteus isolated membranes, C6H was found not to inhibit the synthesis of lipid II (data not shown). The effect of C6H on peptidoglycan synthesis via binding to lipid II was then tested. Using nisin as a positive control, we found that C6H had no apparent capacity to bind lipid II or to inhibit the activity of S. aureus PBP2, which polymerizes lipid II (data not shown).

### C6H affects the proton motive force.

Fig. 1B demonstrates that S. aureus is more susceptible to C6H at low pH. At pH 5.5, C6H of 3 μg/ml leads to cell death (Fig. 1C), but without apparent loss of membrane integrity (Fig. 2A). The action of C6H may involve movement of protons across the membrane, with C6H acting as a protonophore. The proton motive force (PMF) has two components, membrane potential (ΔΨ) and proton gradient (ΔpH) (PMF,Δp = ΔΨ − 59 ΔpH). The cells use both to generate ATP, using the flow of protons through the ATPase. A protonophore affects both components of the PMF. At high concentrations of C6H (≥5 μg/ml), the membrane depolarizes rapidly to become unpolarized (data not shown). Lower concentrations of C6H (3 μg/ml) led to an apparent membrane depolarization (Fig. 2C) and then slower repolarization than with valinomycin. Hence, C6H affects the ΔΨ component of the PMF.

**FIG 3:**
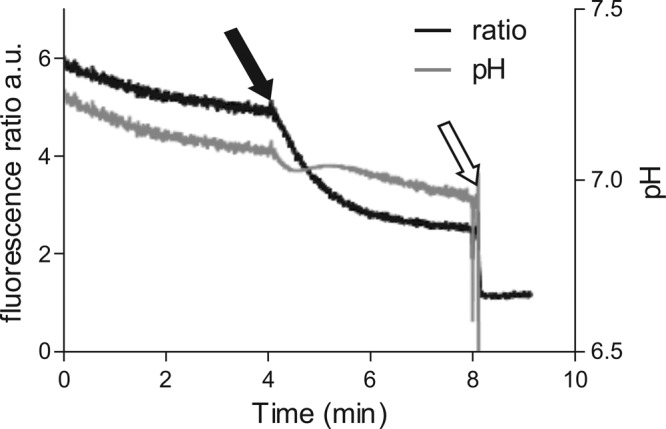
C6H reduces the ability to control internal pH in S. aureus. Effect of C6H on fluorescence ratio of cFDASE (left ordinate) and corresponding pH (right ordinate). C6H (3 μg/ml) was added (filled arrow), followed by excess amounts of nigericin and valinomycin (white arrow).

The ΔpH was measured using the fluorophore cFDASE. Intracellularly, the ratio of the emissions at 530 nm for excitation levels at 500 and 440 nm is a marker of pH. By calibrating that ratio it is then possible to assess the intracellular pH. High concentrations of C6H (≥5 μg/ml) led to a rapid drop of internal pH within minutes, resulting in an equilibration of the internal pH with the outside pH of 5.5 (data not shown). With 3 μg/ml C6H, the internal pH drops rapidly by around 0.1 pH unit in 2 min and then stabilizes (Fig. 3). It appears that at 3 μg/ml C6H, protons are being carried into the cell even though the membrane is still intact. Thus, C6H has protonophore activity and at high concentrations can act as a likely surfactant.

### C6H reduces internal ATP levels.

Two minutes after the addition of a high concentration of C6H (≥5 μg/ml), the ATP level inside the cell drops by 85%, while the percentage of ATP outside cells increases by ∼40% (data not shown). This is consistent with the damage to the membrane, which allows ATP to be released. With 3 μg/ml C6H the cells lose around 93% ATP within 2 min of incubation (Table 1), while the ATP outside the cell increases only by ∼10% compared to the untreated sample (data not shown). The drop in ATP levels may be due to the use of energy by the cell to restore the ΔΨ and ΔpH and/or cessation of ATP synthesis due to loss of the proton gradient required for its production.

**TABLE 1:** Effects of inhibitors on intracellular ATP levels^*a*^

C6H (μg/ml)	ATP level (mean ± SD) (%) (*P*) after 2-min incubation with:			
	No inhibitor	20 μg/ml DCCD	0.5 μg/ml CCCP	10 μg/ml valinomycin
0	96.4 ± 10.9	84.7 ± 12.9 (0.58)	17.8 ± 6.3 (<0.01)	78.9 ± 28.9 (0.49)
3	6.7 ± 0.3	68.8 ± 11.9 (<0.01)	3.2 ± 0.3 (0.01)	46.2 ± 3.2 (<0.01)

a Cells were incubated for 2 min in 20 mM MES (pH 5.5) in the presence or absence of C6H and different inhibitors. ATP levels were determined (expressed as a percentage of the level at 0 min). *P* values are shown for the comparison of inhibitor-treated samples to their respective controls (with or without C6H).

### Effect of C6H in combination with other inhibitors.

In order to further elucidate the potential mechanism of C6H activity, its effects were determined in combination with inhibitors of known function. The protonophore carbonyl cyanide *m*-chloro phenyl hydrazone (CCCP) is a very powerful uncoupling agent that carries protons across the membrane, resulting in loss of both ΔpH and ΔΨ (37). C6H and CCCP have an additive effect on S. aureus killing (Table 2), with an associated drop in ATP levels (Table 1).

**TABLE 2:** Effect of C6H and inhibitors on survival of S. aureus^*a*^

Addition	Survival (mean ± SD) (%) at 90 min compared to untreated control for:					
	Control	50 mM KCl	20 μg/ml DCCD	0.5 μg/ml CCCP	10 μg/ml valinomycin	10μg/ml valinomycin, 50 mM KCl
None	100	91.5 ± 10.6	12.9 ± 3.5	32.2 ± 6.6	2.1 ± 0.1	35.8 ± 5.1
3 μg/ml C6H	7.4 ± 1.8	26.5 ± 6.7	10.1 ± 3.2	1.1 ± 0.5	0.06 ± 0.05	8.6 ± 0.8

a Cells were incubated in the presence or absence of C6H, inhibitors, and KCl for 90 min. All values are expressed as the percent survival compared to the untreated control at 90 min. KCl greatly enhanced survival in the presence of C6H, with or without valinomycin (*P* < 0.01 compared to no-KCl control in both cases).

Valinomycin is a potassium carrier which equilibrates intra- and extracellular levels. It affects ΔΨ and can either depolarize or hyperpolarize the cells, depending on the extracellular K^+^ levels. When valinomycin was used alone, this resulted in only a small loss of ATP, and when used in combination it prevented the drastic loss seen with C6H alone (Table 1). Valinomycin leads to membrane hyperpolarization, in contrast to C6H (Fig. 2C), and with valinomycin and C6H in combination, the membrane becomes transiently hyperpolarized for 5 min prior to a slow return to control levels. With the combination of inhibitors there is also an additive effect in terms of S. aureus killing (Table 2). Addition of KCl significantly reduces the bactericidal effect of both C6H and valinomycin (Table 2).

Dicyclohexylcarbodiimide (DCCD) is a direct inhibitor of ATPase and affects the ΔpH. DCCD caused significant death of S. aureus (Table 2), but when DCCD was used in combination with C6H there was no increase in killing. In addition, DCCD alone resulted in only a small loss of ATP, and in combination with C6H more ATP was maintained than with C6H alone (Table 1). DCCD inhibits the activity of ATPase, thus preventing the use of ATP while extruding protons. As DCCD-treated cells did not lose a significant amount of ATP under the conditions used with 20 mM MES (pH 5.5) (Table 1) (*P* = 0.58), it appears that the cells are not metabolically active under these conditions.

### C6H inhibits the electron transport chain.

**FIG 4:**
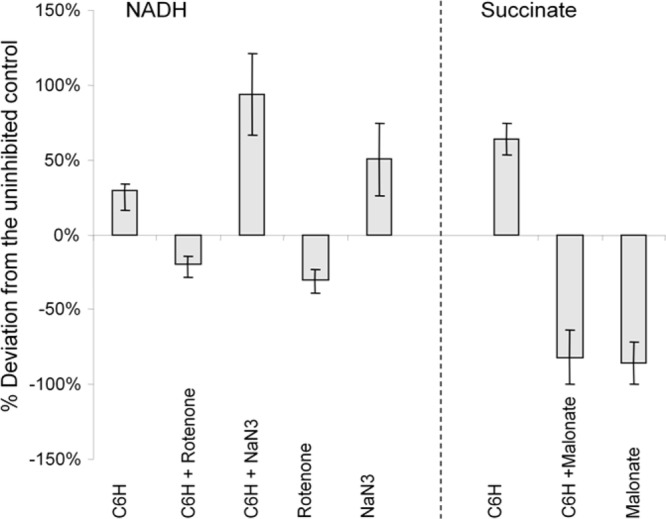
Effect of C6H and various inhibitors on the electron transport chain of E. coli inverted vesicles. NADH (left) and succinate (right) were used as the substrates to generate the electron flow. Data are expressed as percent deviations in formazan production from substrates alone using the INT reduction assay.

The effect of C6H on the electron transport chain (ETC) was studied using inverted vesicles and iodonitrotetrazolium chloride (INT) (28). INT is reduced by almost all the components of the ETC, forming iodonitrotetrazolium violet formazan (INF), which can be colorimetrically quantified. NADH or succinate substrate was added to generate the electron flow. Inhibition of the ETC downstream of the site of INT reduction, i.e., closer to the terminal oxidase (complex IV), should either increase or not affect the amount of formazan compared to control (28). The effect of C6H on the ETC with Escherichia coli inverted vesicles is shown in Fig. 4 and was confirmed with *Micrococcus flavus* inverted vesicles (data not shown). Inhibition of the first components of the ETC, complex I by rotenone in the NADH reaction and complex II by malonate when succinate was used as the substrate, resulted in a decrease of INF formed, whereas inhibition of complex IV by sodium azide resulted in an increased amount of INF (Fig. 4). Similar to NaN_3_, C6H produced larger amounts of formazan. However, no effect of the C6H was seen in the absence of an electron flow, for instance when used in combination with rotenone or malonate. Moreover, a cumulative effect in combination with NaN_3_ was observed, suggesting that C6H interferes with the electron flow through the ETC. Confirmation will require the establishment of the assays using the native S. aureus system.

### Effect of C6H on external ion levels.

Ion chromatography (IC) was used to determine the effects of inhibitors (C6H and CCCP) on ion levels. Of the extracellular ions tested, all were unchanged (data not shown) in the presence of inhibitors (apart from K^+^). As expected, CCCP leads to K^+^ extrusion (6.49 ± 0.02 and 2.67 ± 0.15 ppm for the CCCP treated and controls, respectively), which most likely occurs during reestablishment of the membrane potential. C6H also results in significant release of K^+^ (4.47 ± 0.14 ppm; *P* < 0.01 compared to the control).

### Anaerobic growth leads to increased C6H resistance.

To test the hypothesis that C6H is a protonophore, the MIC for C6H in chemically defined medium under aerobic and anaerobic conditions was tested. Anaerobically grown S. aureus is not dependent on the PMF for survival. Anaerobically grown cells have an MIC of 60 μg/ml, around 6-fold higher than aerobically grown cells (10 μg/ml).

### C6H inhibits respiration.

The addition of half-MIC C6H (5 μg/ml) to S. aureus in TSB iron limited led to a 6-fold reduction in respiration rates (0.09 ± 0.01 and 0.58 ± 0.13 μmol O_2_/ml · s for C6H treated and control, respectively; *P* < 0.0001), whereas 5 μg/ml CCCP (10× MIC) had no significant effect (0.56 ± 0.07 μmol O_2_/ml · s; *P* = 0.83).

### C6H increases membrane fluidity.

1,6-Diphenyl-1,3,5-hexatriene (DPH) is a hydrophobic fluorophore which intercalates between the phospholipids of the bacterial membrane and orients perpendicularly to the membrane plane. The fluorescence of DPH depends on its environment and increases with hydrophobicity. It predominantly reflects the structural order of membrane lipids, mainly as a result of preferential partitioning of the probe into the hydrocarbon phase of the lipid bilayer membranes. Thus, membrane fluidity may be considered the reciprocal of the structured order of membrane lipids. The polarization index value reflects membrane fluidity. An increase of the polarization index equates to an increase in membrane rigidity, while a decrease of the polarization index correlates with increased membrane fluidity.

**FIG 5:**
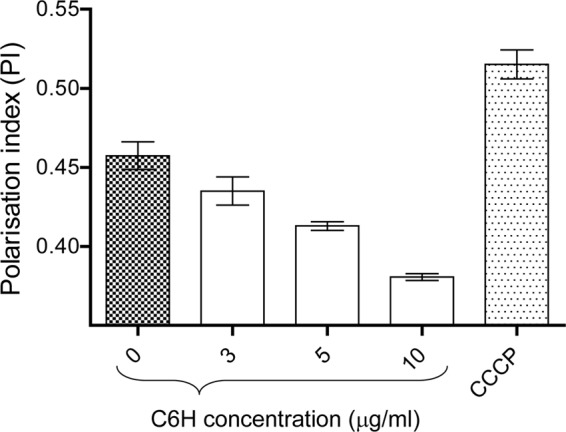
C6H increases S. aureus membrane fluidity. Polarization index (PI) dynamics were measured after treatment of S. aureus cells with C6H or CCCP (0.5 μg/ml).

Addition of C6H led to a decreased polarization index of the bacteria as a result of increased membrane fluidity (Fig. 5). In contrast, CCCP resulted in an increase in membrane rigidity (Fig. 5). This is possible evidence that C6H localizes to the membrane of S. aureus cells. Thus, although both C6H and CCCP are protonophores, they have different mechanisms of action.

### Action of C6H in liposomes.

Liposomes that held the fluorophore carboxyfluorescein (for which the fluorescence is pH dependent) were used to test the effect of Na^+^ and K^+^ ions on the C6H effect. The liposome inclusion had a basic pH due to the solubilization of the fluorophore in sodium hydroxide, which gave a background stable fluorescence. Liposomes also included either 50 mM KCl or 50 mM NaCl. Addition of 50 μg/ml C6H (the concentration needed to see an effect in this artificial model) led to a drop in fluorescence synonymous with a drop of pH inside the liposomes (5.09 ± 2.5 and 0.69 ± 0.6 absorbance units [AU]/min, respectively, for KCl- and NaCl-filled liposomes). Interestingly, the rate of loss of fluorescence is greater with KCl in the liposomes (*P* = 0.01). This suggests that C6H carries protons into the liposomes and then is flipped back with a K^+^ ion. This would allow C6H to then transfer another proton and so increase the rate of fluorescence drop by DPH.

### Cell morphology.

**FIG 6:**
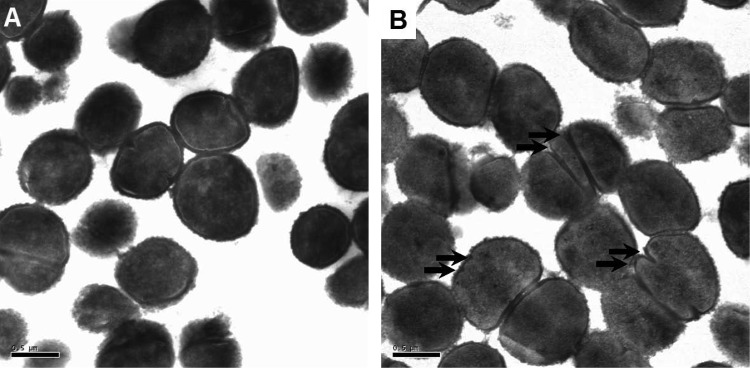
S. aureus morphology in the presence of C6H. Transmission electron microscopy was used to investigate the morphology of S. aureus after a 2-h standard killing assay in the presence (B) or absence (A) of 5 μg/ml C6H. Scale bars, 0.5 μm. The arrows highlight aberrant septation events.

C6H increases membrane fluidity, reduces ATP levels, and leads to cell death. Electron microscopy (EM) was used to determine if C6H morphological changes occur. After a 2-h killing assay with or without 5 μg/ml C6H, samples were prepared for EM imaging. During this time many cells had developed multiple and aberrant positioning of the septa (Fig. 6), possibly as a result of membrane damage. At 3 μg/ml C6H, no significant morphological changes were apparent (data not shown).

### Antimicrobial activity of C6H analogues.

**FIG 7:**
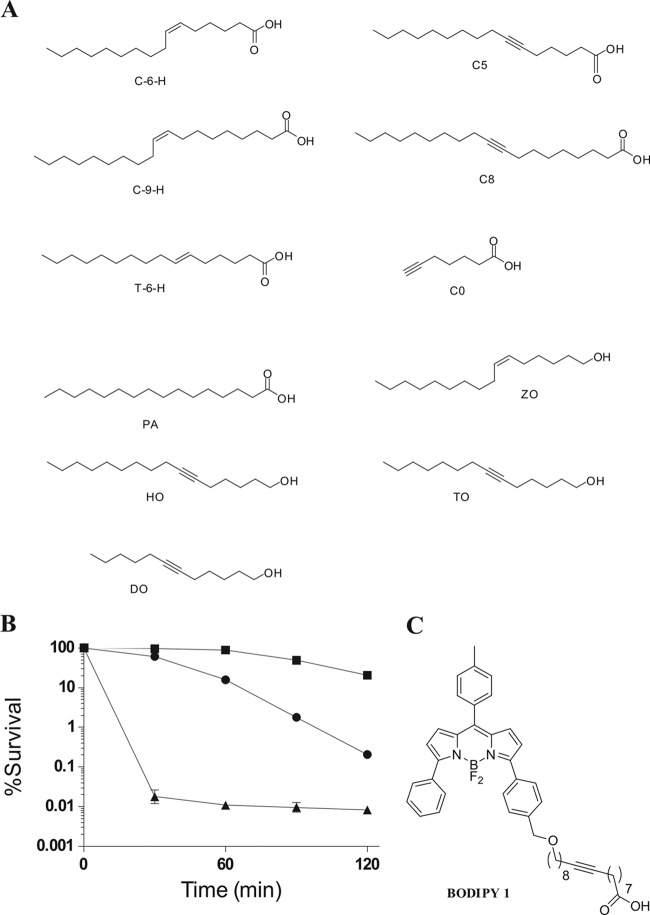
Bactericidal activity of C6H analogues. (A) Various analogues of C6H of the structure shown were chemically synthesized and purified. (B) Antibacterial activity of C6H (●), C8 (▲), and a fluorescent derivative, Bodipy 1 (■). For all data points, the percent survival was compared to that of the wild type at the same time. All compounds were at 17 μM (equivalent to 5 μg/ml C8). ■, Bodipy; ●, C6H; ▲, C8. (C) Structure of Bodipy 1.

In order to begin to define the structure-function relationship for C6H, and the potential for novel antimicrobials, a number of analogues were synthesized. A number of alkynyl acids and alcohols were synthesized and tested for activity (Fig. 7 and data not shown). All alkynyl/alkenyl alcohols were biologically inactive against S. aureus; thus, the polar nature of the carboxylic acid group is important in the bactericidal activity of C6H. A degree of fatty acid unsaturation is required, as palmitic acid, the completely saturated analogue of C6H, showed minimal activity. Interestingly, the *trans* isomer of C6H was not as biologically active against S. aureus as the *cis* isomer. However, alkynyl (triple-bond) analogues of C6H also showed killing similar to C6H levels, further reinforcing the hypothesis that unsaturation of the acid is important for activity (data not shown).

**FIG 8:**
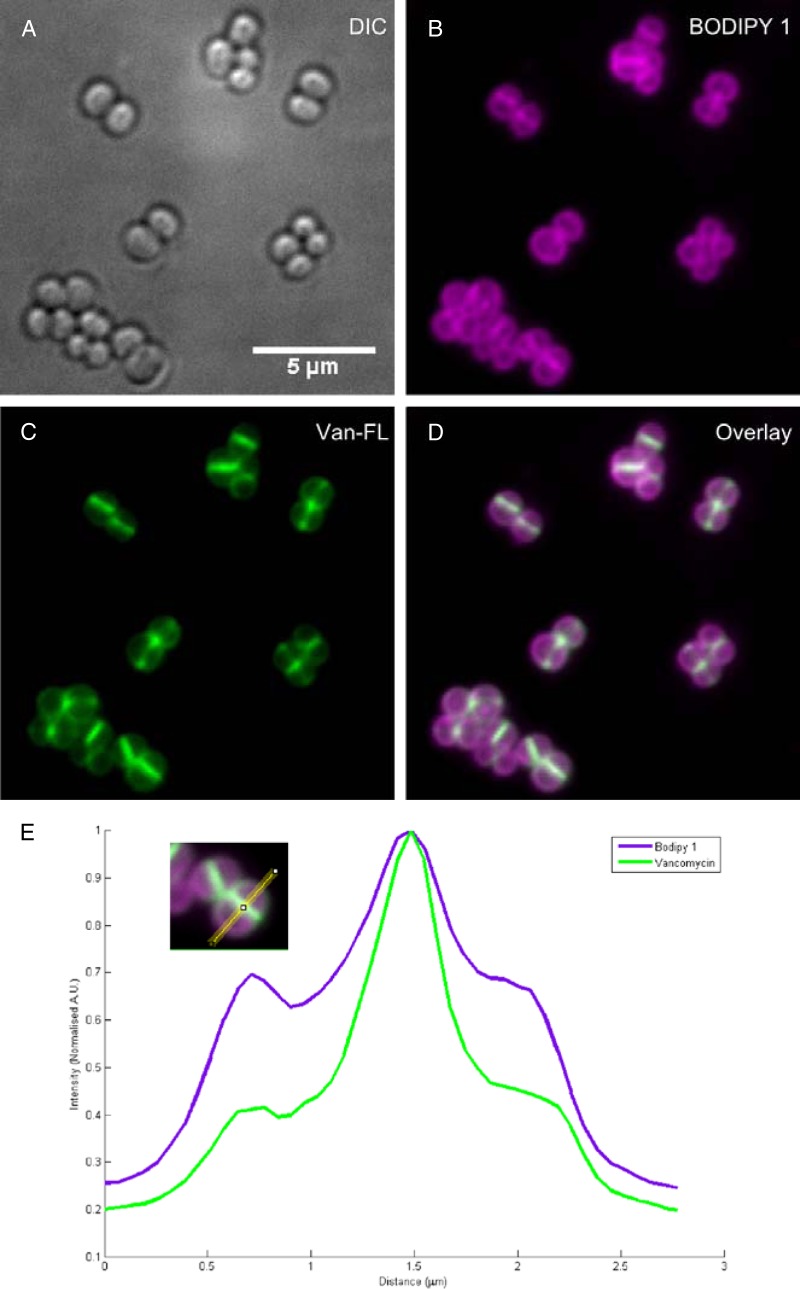
Subcellular localization of Bodipy 1. S. aureus SH1000 was treated with FL-vancomycin and Bodipy 1, washed, and visualized by fluorescence microscopy. (A) DIC; (B) Bodipy 1 labeling; (C) FL-vancomycin labeling; (D) overlay of panels B and C. (E) Line scan through the cell confirming the overall staining of Bodipy 1 labeling throughout the cell membrane.

Interestingly, one of the synthetic analogues (C8) has a higher efficacy against S. aureus than C6H (Fig. 7). This compound was used to develop a fluorescent derivative for localization studies (Bodipy 1). Bodipy 1 still retained some antibacterial activity compared to an untreated control (Fig. 7). The reduction in the killing ability of Bodipy 1 compared to alkynyl acid C8 is attributable to the increase in size of the antimicrobial agent. Bodipy 1 was then used to determine its localization during treatment of S. aureus, using vancomycin as a control for cell wall localization (32). Vancomycin (Fig. 8) shows the characteristic labeling with a focus at the septum, where cell wall synthesis is occurring. Bodipy 1 is an excellent fluorophore, with exceptional brightness at the concentration used (Fig. 8). Bodipy 1 also partitions into the cell envelope and does not accumulate in the cytoplasm (Fig. 8). Bodipy 1 does not demonstrate the pronounced septal localization of vancomycin and partitions more consistently throughout the membrane of S. aureus (Fig. 8e).

## DISCUSSION

Human skin fatty acids are an important facet of innate immunity. In particular, C6H has been found to be the most effective antistaphylococcal fatty acid on human skin (7). Altered fatty acid metabolism and consequent reduction in C6H levels correlates with cases of atopic dermatitis and increased colonization by S. aureus (7). In fact, purified C6H is used to treat atopic dermatitis and reduce S. aureus levels (7). Thus, if one is to develop C6H or related molecules as potential prophylaxis or therapy, its mode of action needs to be established. The bactericidal activity of fatty acids requires them to be in the acid form (the ester form does not kill), to have a length of >14 carbons, and to be unsaturated (38, 39, 40, 41). C6H is a 16-carbon, monounsaturated fatty acid (42, 43) made by the sebaceous glands from the widely available saturated palmitic acid (44). We have found that there are specific functional group characteristics that are essential for the activities of these molecules. Whether this effect is through a direct molecular interaction of the unsaturation with a key target in the cell membrane, or through changes induced in the shape of these molecules at the macromolecular level, is not clear. Further studies aimed at examining the key roles of the pharmacophores in these fatty acids are currently being conducted.

Although several bactericidal mechanisms of fatty acid activity have been postulated, they have remained elusive. We propose that C6H kills S. aureus in several ways. It is also possible that subpopulations of the cells may exhibit altered susceptibility to C6H. All mechanisms are linked and require C6H to access and partition into the bacterial membrane. In support of this, analysis of the fluorescent analogue Bodipy 1 demonstrated cell envelope localization for this compound. At low concentrations, dependent on the physicochemical environment and the metabolic status of the cells, C6H increases membrane fluidity and has a protonophore effect. C6H leads to a loss of membrane potential and an inability to control internal pH. This explains why C6H is more effective in killing S. aureus at the acidic pH of the skin (pH 5.5). The acidic group on the fatty acid C6H is likely essential for its proposed protonophore effect. The ionized negatively charged acidic group COO^−^ at low pH may be necessary to coordinate a positive proton H^+^. Inside the cells, the proton is released in the neutral pH cytoplasm. CCCP is also a protonophore (37), and simultaneous application of both compounds leads to an increased bactericidal effect.

The synergy of C6H and valinomycin suggests that the proton transfer by C6H is partly dependent on the membrane charge, i.e., membrane potential itself. C6H transports protons from the outside of the cell to the inside, while valinomycin carries potassium ions from the outside to the inside, seemingly reestablishing the membrane potential and thus allowing C6H to carry more protons. It also appears that the rate of proton transport by C6H is slower than the rate of potassium transport by valinomycin, because when both are used together the cells are being hyperpolarized, although to a lesser extent than when valinomycin is used on its own. However, when potassium is present outside the cell, the synergy between C6H and valinomycin is lost. Salts have inhibitory effects on the bactericidal activity of C6H, which might occur because salts may stabilize the membrane or bind to C6H, altering its activity.

C6H, unlike CCCP, may not be proton specific and can potentially carry other ions. This provides a potential mechanism to explain how C6H, after transferring a proton across the membrane, can flip back, carrying another ion, and then transfer a further proton. IC and liposome data suggest that this secondary ion would be K^+^. CCCP also generates a release of potassium ions, similarly to nigericin. Cells use the export of potassium ions to reestablish the membrane potential destroyed by CCCP action (45, 46). However, the liposome data suggest that C6H can carry K^+^ in either direction. Such a nonspecific transport has been previously described in regard to the effect of various fatty acids on liposomes (35, 47). This may also explain why, after the initial depolarization generated by C6H, cells repolarize their membrane within 20 min (Fig. 2). Thus, C6H may transport protons and K^+^ across the cell membrane, with the extent and direction of transfer being determined by the external and internal environments. Ion movements generated by C6H flipping across the membrane might be dependent on a specific protein. Such a mechanism is present in mitochondria of young mammals, allowing uncoupling of the respiratory chain (40, 48, 49). The artificial liposomes used here suggest that C6H can act independently of protein interactions.

A central question remains as to how C6H actually kills S. aureus. At concentrations too low to cause overt membrane disruption, it is not solely the loss of membrane potential (such as when C6H is used in combination with valinomycin and Δψ remains stable), that leads to increased mortality. In addition, intracellular ATP levels are better preserved when C6H and valinomycin are used together. The drop in the internal pH level is likely to affect cell survival, as numerous enzymatic processes are pH dependent within the cells. Another factor that is likely to determine cell viability is the ability of C6H to induce a clear increase in membrane fluidity. This may lead to uncoupling of the respiratory chain and disruption of membrane-associated processes leading to cell division defects, as observed by electron microscopy. Furthermore, studies performed in mitochondria showed that fatty acids modulate mitochondrial reactive oxygen species (ROS) generation by uncoupling and interfering with electron transport and also by increasing membrane fluidity (50). Interference of fatty acids with mitochondrial electron transport is well documented (51, 52), although its molecular mechanism is not well understood (50).

Thus, C6H has multiple physiological consequences for S. aureus, and previous work has shown dramatic effects on gene expression (53) and an ability to inhibit virulence factor production (9). C6H has also been found to be useful in the treatment of both topical and systemic S. aureus infections (9). This highlights the potential of C6H as a novel therapy. C6H is included in several topical human preparations as an emollient (U.S. patent application 20110097292) and so does not have serious toxicity issues. Chemical synthesis of various C6H analogues has not only begun to identify important features of the molecule, such as the polar carboxylic acid group, alkenyl/alkynyl moiety, and long hydrocarbon chain, but has also highlighted leads for the development of novel molecules to combat such an important pathogen.

## References

[1] Ong PY Ohtake T Brandt C Strickland I Boguniewicz M Ganz T Gallo RL Leung DY 2002 Endogenous antimicrobial peptides and skin infections in atopic dermatitis. N. Engl. J. Med. 347:1151–1160. 10.1056/NEJMoa021481 12374875

[2] Niyonsaba F Ogawa H 2005 Protective roles of the skin against infection: implication of naturally occurring human antimicrobial agents beta-defensins, cathelicidin LL-37 and lysozyme. J. Dermatol. Sci. 40:157–168. 10.1016/j.jdermsci.2005.07.009 16150577

[3] Wille JJ Kydonieus A 2003 Palmitoleic acid isomer (C16:1delta6) in human skin sebum is effective against Gram-positive bacteria. Skin Pharmacol. Appl. Skin Physiol. 16:176–187. 10.1159/000069757 12677098

[4] Korting HC Lukacs A Braun-Falco O 1988 Microbial flora and odor of the healthy human skin. Hautarzt 39:564–568 (In German.) 3053532

[5] Kluytmans JA Wertheim HF 2005 Nasal carriage of Staphylococcus aureus and prevention of nosocomial infections. Infection 33:3–8. 10.1007/s15010-005-4012-9 15750752

[6] Miller SJ Aly R Shinefeld HR Elias PM 1988 *In vitro* and *in vivo* antistaphylococcal activity of human stratum corneum lipids. Arch. Dermatol. 124:209–215 3341800

[7] Takigawa H Nakagawa H Kuzukawa M Mori H Imokawa G 2005 Deficient production of hexadecenoic acid in the skin is associated in part with the vulnerability of atopic dermatitis patients to colonization by Staphylococcus aureus. Dermatology 211:240–248. 10.1159/000087018 16205069

[8] Georgel P Crozat K Lauth X Makrantonaki E Seltmann H Sovath S Hoebe K Du X Rutschmann S Jiang Z Bigby T Nizet V Zouboulis CC Beutler B 2005 A toll-like receptor 2-responsive lipid effector pathway protects mammals against skin infections with Gram-positive bacteria. Infect. Immun. 73:4512–4521. 10.1128/IAI.73.8.4512-4521.2005 16040962 PMC1201198

[9] Clarke SR Mohamed R Bian L Routh AF Kokai-Kun JF Mond JJ Tarkowski A Foster SJ 2007 The Staphylococcus aureus surface protein IsdA mediates resistance to innate defenses of human skin. Cell Host Microbe 1:199–212. 10.1016/j.chom.2007.04.005 18005699

[10] Dye ES Kapral FA 1981 Characterization of a bactericidal lipid developing within staphylococcal abscesses. Infect. Immun. 32:98–104 7216498 10.1128/iai.32.1.98-104.1981 PMC350593

[11] Kohler T Weidenmaier C Peschel A 2009 Wall teichoic acid protects Staphylococcus aureus against antimicrobial fatty acids from human skin. J. Bacteriol. 191:4482–4484. 10.1128/JB.00221-09 19429623 PMC2698495

[12] Greenway DL Dyke KG 1979 Mechanism of the inhibitory action of linoleic acid on the growth of Staphylococcus aureus. J. Gen. Microbiol. 115:233–245. 10.1099/00221287-115-1-233 93615

[13] Galbraith H Miller TB 1973 Effect of long-chain fatty acids on bacterial respiration and amino acid uptake. J. Appl. Bacteriol. 36:659–675. 10.1111/j.1365-2672.1973.tb04151.x 4787613

[14] Greenway DL Dyke KG 1980 Isolation and properties of a linoleic acid-resistant mutant of Staphylococcus aureus. J. Gen. Microbiol. 118:267–270 7420054 10.1099/00221287-118-1-267

[15] Memon RA Staprans I Noor M Holleran WM Uchida Y Moser AH Feingold KR Grunfeld C 2000 Infection and inflammation induce LDL oxidation *in vivo*. Arterioscler. Thromb. Vasc. Biol. 20:1536–1542. 10.1161/01.ATV.20.6.1536 10845869

[16] Klomsiri C Panmanee W Dharmsthiti S Vattanaviboon P Mongkolsuk S 2005 Novel roles of ohrR-ohr in Xanthomonas sensing, metabolism, and physiological adaptive response to lipid hydroperoxide. J. Bacteriol. 187:3277–3281. 10.1128/JB.187.9.3277-3281.2005 15838057 PMC1082813

[17] Hussain M Hastings JG White PJ 1991 A chemically defined medium for slime production by coagulase-negative staphylococci. J. Med. Microbiol. 34:143–147. 10.1099/00222615-34-3-143 2010904

[18] Ganzle MG Vogel RF 2003 Studies on the mode of action of reutericyclin. Appl. Environ. Microbiol. 69:1305–1307. 10.1128/AEM.69.2.1305-1307.2003 12571063 PMC143594

[19] Schneider T Senn MM Berger-Bachi B Tossi A Sahl HG Wiedemann I 2004 *In vitro* assembly of a complete, pentaglycine interpeptide bridge containing cell wall precursor (lipid II-Gly5) of Staphylococcus aureus. Mol. Microbiol. 53:675–685. 10.1111/j.1365-2958.2004.04149.x 15228543

[20] Rick PD Hubbard GL Kitaoka M Nagaki H Kinoshita T Dowd S Simplaceanu V Ho C 1998 Characterization of the lipid-carrier involved in the synthesis of enterobacterial common antigen (ECA) and identification of a novel phosphoglyceride in a mutant of Salmonella Typhimurium defective in ECA synthesis. Glycobiology 8:557–567. 10.1093/glycob/8.6.557 9592122

[21] Martinez B Bottiger T Schneider T Rodriguez A Sahl HG Wiedemann I 2008 Specific interaction of the unmodified bacteriocin Lactococcin 972 with the cell wall precursor lipid II. Appl. Environ. Microbiol. 74:4666–4670. 10.1128/AEM.00092-08 18539790 PMC2519333

[22] Kohlrausch U Holtje JV 1991 One-step purification procedure for UDP-*N*-acetylmuramyl-peptide murein precursors from Bacillus cereus. FEMS Microbiol. Lett. 62:253–257 1904044 10.1016/0378-1097(91)90166-8

[23] Raafat D von Bargen K Haas A Sahl HG 2008 Insights into the mode of action of chitosan as an antibacterial compound. Appl. Environ. Microbiol. 74:3764–3773. 10.1128/AEM.00453-08 18456858 PMC2446574

[24] Breeuwer P Drocourt J Rombouts FM Abee T 1996 A novel method for continuous determination of the intracellular pH in bacteria with the internally conjugated fluorescent probe 5 (and 6-)-carboxyfluorescein succinimidyl ester. Appl. Environ. Microbiol. 62:178–183 16535209 10.1128/aem.62.1.178-183.1996 PMC1388751

[25] Bayer AS Prasad R Chandra J Koul A Smriti M Varma A Skurray RA Firth N Brown MH Koo SP Yeaman MR 2000 *In vitro* resistance of Staphylococcus aureus to thrombin-induced platelet microbicidal protein is associated with alterations in cytoplasmic membrane fluidity. Infect. Immun. 68:3548–3553. 10.1128/IAI.68.6.3548-3553.2000 10816510 PMC97641

[26] Camargo IL Neoh HM Cui L Hiramatsu K 2008 Serial daptomycin selection generates daptomycin-nonsusceptible Staphylococcus aureus strains with a heterogeneous vancomycin-intermediate phenotype. Antimicrob. Agents Chemother. 52:4289–4299. 10.1128/AAC.00417-08 18824611 PMC2592861

[27] Mishra NN Yang SJ Sawa A Rubio A Nast CC Yeaman MR Bayer AS 2009 Analysis of cell membrane characteristics of *in vitro*-selected daptomycin-resistant strains of methicillin-resistant Staphylococcus aureus. Antimicrob. Agents Chemother. 53:2312–2318. 10.1128/AAC.01682-08 19332678 PMC2687258

[28] Smith JJ McFeters GA 1997 Mechanisms of INT (2-(4-iodophenyl)-3-(4-nitrophenyl)-5-phenyl tetrazolium chloride), and CTC (5-cyano-2,3-ditolyl tetrazolium chloride) reduction in Escherichia coli K-12. J. Microbiol. Methods 29:161–175. 10.1016/S0167-7012(97)00036-5 8642015

[29] Burstein C Tiankova L Kepes A 1979 Respiratory control in Escherichia coli K-12. Eur. J. Biochem. 94:387–392. 10.1111/j.1432-1033.1979.tb12905.x 371964

[30] Bonelli RR Schneider T Sahl HG Wiedemann I 2006 Insights into in vivo activities of lantibiotics from gallidermin and epidermin mode-of-action studies. Antimicrob. Agents Chemother. 50:1449–1457. 10.1128/AAC.50.4.1449-1457.2006 16569864 PMC1426925

[31] Fu Y Weng Y Hong WX Zhang Q 2010 Efficient synthesis of unsaturated 1-monoacyl glycerols for in meso crystallization of membrane proteins. Synlett 2011:809–812. 10.1055/s-0030-1259912 21461138 PMC3068204

[32] Watson SP Clements MO Foster SJ 1998 Characterization of the starvation-survival response of Staphylococcus aureus. J. Bacteriol. 180:1750–1758 9537371 10.1128/jb.180.7.1750-1758.1998 PMC107086

[33] Turner RD Ratcliffe EC Wheeler R Golestanian R Hobbs JK Foster SJ 2010 Peptidoglycan architecture can specify division planes in Staphylococcus aureus. Nat. Commun. 1:26. 10.1038/ncomms1025 20975691

[34] Levin J Maibach H 2008 Human skin buffering capacity: an overview. Skin Res. Technol. 14:121–126. 10.1111/j.1600-0846.2007.00271.x 18412552

[35] Schonfeld P Gerke S Bohnensack R Wojtczak L 2003 Stimulation of potassium cycling in mitochondria by long-chain fatty acids. Biochim. Biophys. Acta 1604:125–133. 10.1016/S0005-2728(03)00043-4 12765769

[36] Bierbaum G Sahl HG 2009 Lantibiotics: mode of action, biosynthesis and bioengineering. Curr. Pharm. Biotechnol. 10:2–18. 10.2174/138920109787048616 19149587

[37] Felle H Bentrup FW 1977 A study of the primary effect of the uncoupler carbonyl cyanide *m*-chlorophenylhydrazone on membrane potential and conductance in *Riccia fluitans*. Biochim. Biophys. Acta 464:179–187. 10.1016/0005-2736(77)90380-7 831789

[38] Willett NP Morse GE 1966 Long-chain fatty acid inhibition of growth of Streptococcus agalactiae in a chemically defined medium. J. Bacteriol. 91:2245–2250 5943940 10.1128/jb.91.6.2245-2250.1966 PMC316201

[39] Butcher GW King G Dyke KG 1976 Sensitivity of Staphylococcus aureus to unsaturated fatty acids. J. Gen. Microbiol. 94:290–296. 10.1099/00221287-94-2-290 950553

[40] Wojtczak L Wieckowski MR 1999 The mechanisms of fatty acid-induced proton permeability of the inner mitochondrial membrane. J. Bioenerg. Biomembr. 31:447–455. 10.1023/A:1005444322823 10653473

[41] Kelsey JA Bayles KW Shafii B McGuire MA 2006 Fatty acids and monoacylglycerols inhibit growth of Staphylococcus aureus. Lipids 41:951–961. 10.1007/s11745-006-5048-z 17180883

[42] Downing DT Strauss JS 1974 Synthesis and composition of surface lipids of human skin. J. Investig. Dermatol. 62:228. 10.1111/1523-1747.ep12676793 5347411

[43] James AT Wheatley VR 1956 Studies of sebum. 6. The determination of the component fatty acids of human forearm sebum by gas-liquid chromatography. Biochem. J. 63:269–273 13328821 10.1042/bj0630269 PMC1216037

[44] Ge L Gordon JS Hsuan C Stenn K Prouty SM 2003 Identification of the delta-6 desaturase of human sebaceous glands: expression and enzyme activity. J. Invest. Dermatol. 120:707–714. 10.1046/j.1523-1747.2003.12123.x 12713571

[45] Cirillo VP 1966 Symposium on bioelectrochemistry of microorganisms. I. Membrane potentials and permeability. Bacteriol. Rev. 30:68–79 5324650 10.1128/br.30.1.68-79.1966 PMC378215

[46] Skulachev VP 1978 Membrane-linked energy buffering as the biological function of Na^+^/K^+^ gradient. FEBS Lett. 87:171–179. 10.1016/0014-5793(78)80326-3 344066

[47] Sharpe MA Cooper CE Wrigglesworth JM 1994 Transport of K^+^ and other cations across phospholipid membranes by nonesterified fatty acids. J. Membr. Biol. 141:21–28 7966242 10.1007/BF00232870

[48] Skulachev VP 1998 Uncoupling: new approaches to an old problem of bioenergetics. Biochim. Biophys. Acta 1363:100–124. 10.1016/S0005-2728(97)00091-1 9507078

[49] Nicholls DG 2001 A history of UCP1. Biochem. Soc. Trans. 29:751–755. 10.1042/BST0290751 11709069

[50] Schonfeld P Wojtczak L 2007 Fatty acids decrease mitochondrial generation of reactive oxygen species at the reverse electron transport but increase it at the forward transport. Biochim. Biophys. Acta 1767:1032–1040. 10.1016/j.bbabio.2007.04.005 17588527

[51] Skulachev VP 1991 Fatty acid circuit as a physiological mechanism of uncoupling of oxidative phosphorylation. FEBS Lett. 294:158–162. 10.1016/0014-5793(91)80658-P 1756853

[52] Di Paola M Lorusso M 2006 Interaction of free fatty acids with mitochondria: coupling, uncoupling and permeability transition. Biochim. Biophys. Acta 1757:1330–1337. 10.1016/j.bbabio.2006.03.024 16697347

[53] Kenny JG Ward D Josefsson E Jonsson IM Hinds J Rees HH Lindsay JA Tarkowski A Horsburgh MJ 2009 The Staphylococcus aureus response to unsaturated long-chain free fatty acids: survival mechanisms and virulence implications. PLoS One 4:e4344. 10.1371/journal.pone.0004344 19183815 PMC2629846

